# Supplementary material for: Diversity-Generating Retroelements in Prokaryotic Immunity
Source: Int J Mol Sci. 2023 Mar 15;24(6):5614. doi: 10.3390/ijms24065614 (PMC10053308; doi:10.3390/ijms24065614)
Supplement: Supplementary file 1 [file ijms-24-05614-s001.zip › ijms-2199665-supplementary.pdf]

Supplementary Materials: Diversity-generating retroelements in prokaryotic immunity

Ilya S. Belalov [1,*], Arseniy A. Sokolov [1,2] and Andrey V. Letarov [1,*]

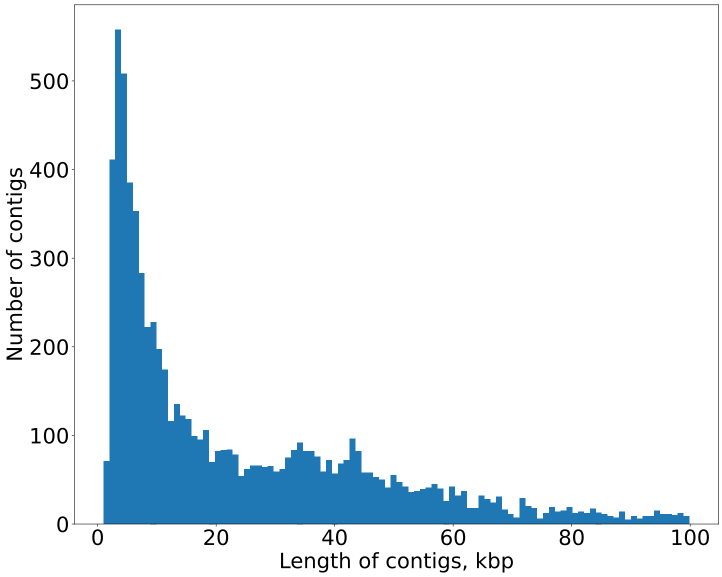

Figure S1. Contig length distributions.

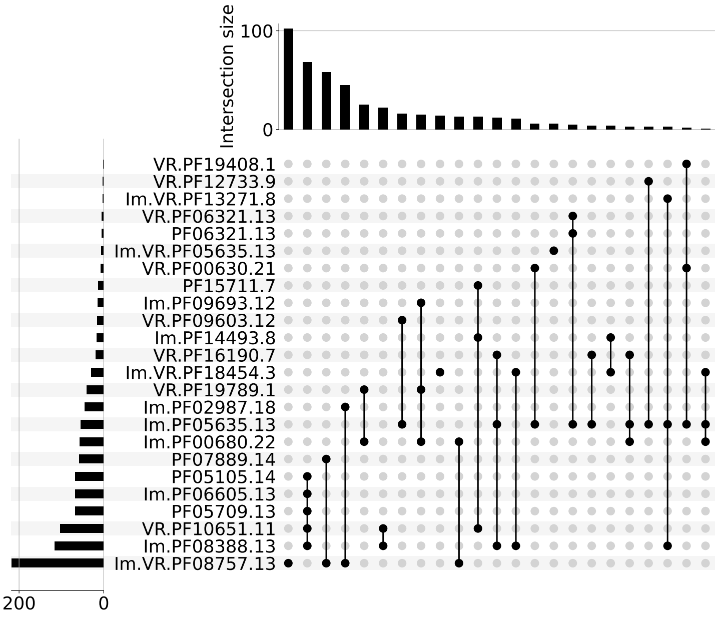

Figure S2. Pfam compositions from Fig. 2 among contigs longer than 20 kbp and for which each Pfam had an Icity score of > 0.7 in all seven runs.

Table S1. Accession codes, names, and short descriptions for Pfams listed in Figure 2 in the main text.

Accession code	Name	Description
PF00028.19	Cadherin	Cadherin domain
PF00536.32	SAM_1	SAM domain (Sterile alpha motif)
PF00570.25	HRDC	HRDC domain
PF00630.21	Filamin	Filamin/ABP280 repeat
PF00680.22	RdRP_1	Viral RNA-dependent RNA polymerase
PF02987.18	LEA_4	Late embryogenesis abundant protein
PF05105.14	Phage_holin_4_1	Bacteriophage holin family
PF05635.13	23S_rRNA_IVP	23S rRNA-intervening sequence protein
PF05709.13	Sipho_tail	Phage tail protein
PF05729.14	NACHT	NACHT domain
PF06321.13	P_gingi_FimA	Major fimbrial subunit protein (FimA)
PF06605.13	Prophage_tail	Prophage endopeptidase tail
PF07889.14	DUF1664	Protein of unknown function (DUF1664)
PF08388.13	GIIM	Group II intron, maturase-specific domain
PF08757.13	CotH	CotH kinase protein
PF09603.12	Fib_succ_major	Fibrobacter succinogenes major domain
PF09693.12	Phage_XkdX	Phage uncharacterised protein (Phage_XkdX)
PF10651.11	BppU_N	BppU N-terminal domain
PF12733.9	Cadherin-like	Cadherin-like beta sandwich domain
PF13271.8	DUF4062	Domain of unknown function (DUF4062)
PF14493.8	HTH_40	Helix-turn-helix domain
PF14870.8	PSII_BNR	Photosynthesis system II assembly factor YCF48
PF15711.7	ILEI	Interleukin-like EMT inducer
PF16190.7	E1_FCCH	Ubiquitin-activating enzyme E1 FCCH domain
PF18454.3	Mtd_N	Major tropism determinant N-terminal domain
PF18676.3	MBG_2	MBG domain (YGX type)
PF19190.2	BACON_2	Viral BACON domain
PF19408.1	PKD_6	PKD-like domain
PF19789.1	DUF6273	Family of unknown function (DUF6273)
